# Engaging U.S. experts in environmental oversight of GE crops as it relates to novel biotechnologies

**DOI:** 10.3389/fbioe.2026.1791551

**Published:** 2026-03-30

**Authors:** Nick Loschin, Catherine E. Sanders, Khara Grieger

**Affiliations:** 1 Department of Applied Ecology, North Carolina State University, Raleigh, NC, United States; 2 North Carolina Plant Sciences Initiative, North Carolina State University, Raleigh, NC, United States; 3 Genetic Engineering and Society Center, North Carolina State University, Raleigh, NC, United States; 4 Department of Agricultural and Human Sciences, North Carolina State University, Raleigh, NC, United States

**Keywords:** biotechnology governance, biotechnology regulation, environmental oversight, Genetic engineering, genome editing

## Abstract

Advances in agricultural biotechnologies, including gene editing and other new genomic techniques, are expanding the range of organisms, traits, and applications being released into the environment. Questions have been raised about the adequacy of the existing U.S. environmental governance frameworks. This study investigates U.S. expert stakeholder perspectives on the environmental oversight of genetically engineered crops and emerging biotechnologies using a qualitative methodology rooted in a systems-thinking approach. Semi-structured interviews were conducted with 16 experts in biotechnology oversight from industry, government, academia, and non-governmental organizations. Transcripts were analyzed thematically to identify perceptions related to strengths, limitations, and future needs of the oversight, especially in the wake of emerging technologies. Findings from the interviews indicate broad confidence in the scientific rigor and environmental safety for established genetic engineering applications, particularly in controlled agricultural contexts, contrary to the authors’ initial expectations. At the same time, concerns were raised about the statutory limitations, regulatory burden, and the capacity of the current oversight system to evaluate more novel biotechnologies. Participants consistently emphasized recommendations for more centralized and proactive governance, risk-proportionate assessments, and adaptive management that is more grounded in the trait and impact of new agricultural biotechnologies. These findings highlight the importance of systems-oriented approaches for environmental oversight that supports innovation while protecting ecological sustainability.

## 1 Introduction

Biotechnology has revolutionized agriculture by enabling the development of genetically engineered (GE) crops with traits that improve yield, enhance pest and disease resistance, and increase herbicide tolerance (Anderson et al., 2019; Kniss, 2018; Datta, 2013). More recently, advancements in new genomic techniques (NGTs), such as gene editing, have introduced cost-effective and precise methods for modifying crops, offering potential solutions to global challenges like food security and climate resilience (Chen et al., 2024; Karavolias et al., 2021; Kumar et al., 2020). Biotechnology tools have expanded the use cases of genetic engineering and the diversity of crops and traits being developed. For example, gene editing can accelerate access to improved varieties for smallholder farmers as well as lead to new developments in both major and minor crops (Pixley et al., 2022). Developers are also pursuing more consumer-oriented products, such as anti-browning mushrooms, seedless blackberries, and less pungent mustard greens that have greater appeal to consumers (Wang et al., 2020; Fister et al., 2024; Karlson et al., 2022). Gene editing is also being used *in lieu* of traditional genetic engineering techniques (e.g., transgenesis) to quickly address spreading diseases in crops such as the banana and cacao (Tripathi et al., 2022; Fister et al., 2018). Biotechnology innovations extend beyond plants, with NGTs being used to improve livestock production and to develop disease-resistant animals (Van Eenennaam, 2025; Whitworth et al., 2016; Cimadori et al., 2025). Further use cases are also being developed with GE microbes and the use of gene drives to address complex agricultural and environmental challenges, including insect and pest pressure (Shams et al., 2024; Bier, 2022). These rapidly evolving applications interact with agricultural, ecological, and socio-political systems that introduce new layers of complexity in which emerging biotechnologies, oversight, and regulation operate.

As novel innovations that involve emerging biotechnologies continue to reshape agricultural practices, there is an urgent need to ensure that regulatory frameworks keep pace with these scientific advancements. Today, current oversight of GE crops in the United States (U.S.) is rooted in the Coordinated Framework for the Regulation of Biotechnology (CFRB), with the Environmental Protection Agency (EPA), the United States Department of Agriculture (USDA), and the Food and Drug Administration (FDA) sharing responsibility for assessing agricultural, environmental, and human health safety considerations under their respective statutes (U.S. OSTP, 1986). At the same time, the existing regulatory structures in the U.S. have been criticized in previous studies for inconsistencies, outdated risk assessment criteria, and a lack of transparency (Wolt and Wolf, 2018; Kuzma and Grieger, 2020), as well as the continuous product *versus* process conversations on whether to regulate on how the product was made or based on the final product (Gould et al., 2022). Among other aspects, such challenges illustrate how biotechnology governance often spans multiple institutional systems and where coordination and alignment across agencies becomes essential. While these concerns apply broadly to biotechnology, they are especially relevant as policy continues to try to adapt to the surge of new traits and products that result from these advancements (NSCEB, 2025).

Honing in on the complexity of potential risks and impacts to the environment in particular, environmental risks remain context-specific and shaped by ecological, agronomic, and socio-environmental interactions–despite decades of research demonstrating that GE crops can be environmentally safe in many respects (NASEM, 2016). Recent scholarship highlights key areas where environmental oversight may be insufficient (Loschin et al., 2025; Noack et al., 2024), noting that environmental impacts are often difficult to assess due to complex direct and indirect impacts involving biodiversity, land-use change, ecological interactions, and long-term ecosystem dynamics (Noack et al., 2024; Caradus, 2023a; NASEM, 2016). This growing body of work underscores the need for expanded assessment parameters, long-term ecological monitoring, and more transparent regulatory processes to inform responsive policy development (Kuzma et al., 2023; Hilbeck et al., 2020; Furgurson et al., 2023; Keiper and Atanassova, 2022; Wei et al., 2024).

Environmental and governance complexities raise important questions about whether current oversight mechanisms are adequate for evaluating emerging biotechnologies. Effective environmental oversight is therefore critical to mitigating potential risks associated with unintended ecological effects and biodiversity impacts (Noack et al., 2024). Additionally, ensuring adequate and comprehensive environmental assessment and oversight may also help secure trust between stakeholders, given the previous public debates related to genetic engineering and biotechnology, particularly in food and agriculture sectors (Cummings et al., 2024; Barnhill-Dilling et al., 2021; Kuzma and Grieger, 2020). Striking a balance between fostering innovation and ensuring environmental safety remains a key challenge for many stakeholders, including those in government, industry, academia, and NGOs, making it essential to evaluate how expert stakeholders perceive the strengths and limitations of current oversight processes and structures.

While existing literature discusses gaps in environmental oversight (e.g., Kuzma, 2018; Noack et al., 2024), few studies have been published that focus on systematically gathering expert stakeholder perspectives on these gaps (Kuzma et al., 2009), with none focused solely on environmental oversight. Given that oversight systems are shaped by diverse actors who may have differing or even conflicting priorities, capturing a range of expert perspectives is essential for understanding how the oversight system functions in practice. With the emergence of a broad array of agricultural biotechnologies (e.g., GE microbes, gene drives), understanding how experts view oversight gaps is crucial, as policies are currently being discussed and in some cases revised (Mundorf et al., 2025; Tachikawa and Matsuo, 2024). This study therefore provides insights on the environmental oversight of GE crops and novel biotechnologies based on U.S. expert stakeholders. Findings may not only help clarify different perspectives related to the adequacy of the current environmental oversight processes for GE crops but results from this work may also highlight recommendations to strengthen assessments and oversight processes useful for US and international contexts.

More specifically, this study engages U.S. expert stakeholders from academia, industry, government, and nonprofit sectors (e.g., organizations that operate at the science–policy interface and routinely participate in governance discussions, or advisory processes related to GE crops) to assess perceived strengths and weaknesses of existing environmental assessment processes In this manuscript, the term environmental assessment is used to be consistent with terminology used by U.S. regulatory agencies to refer broadly to environmental evaluation and oversight for GE crops, while reserving environmental risk assessment specifically for the formal, structured process of risk characterization within that broader oversight framework. It also explores expert recommendations for improving oversight, with particular attention to regulatory clarity, risk assessment methodologies, and transparency. Given the increasing complexity of biotechnology applications in agriculture, understanding these perspectives is important to have critical dialogues, better understand diverse stakeholder viewpoints and help shape policies that balance innovation with environmental sustainability (Kuzma and Grieger, 2020). By situating stakeholder perspectives within the broader oversight system, this study offers insight into how governance components interact in relation to environmental safety and informs potential future regulatory refinements. Further, this study adds meaningful contributions to discussions about regulatory oversight, particularly by bringing U.S. expert perspectives into a space where policy decisions are complex and, on occasion, may not be perceived as thoroughly transparent to all stakeholders. The findings may be particularly relevant for policymakers, regulatory agencies, and researchers seeking to improve the governance of emerging biotechnologies in agriculture. In the sections that follow, we describe our qualitative approach, present key themes from stakeholder interviews, and discuss implications for the future of environmental oversight for agricultural biotechnologies.

## 2 Methods

### 2.1 Theoretical approach - systems thinking

The research study utilized a systems thinking approach to inform the development of the research design, interview questions, and data analysis. Systems thinking is a broad multifaceted framework, encompassing various definitions and applications across the literature (Arnold and Wade, 2015; Cabrera et al., 2023; Sanders et al., 2021). At its core, it facilitates the examination of complex systems by emphasizing the interrelationships and boundaries that shape them. As it relates to the environment, a recent National Academies of Sciences, Engineering, and Medicine (NASEM) report has defined systems thinking as “a holistic, scientific approach to environmental issues that involves consideration of complex interactions and feedback between the environment and society at multiple spatial and temporal scales, often across multiple levels of organization from molecules to organisms, populations, communities, and ecosystems” (NASEM, 2023). This definition underscores the relevance of systems thinking in addressing interdisciplinary environmental challenges posed by agricultural innovation and governance policies and practices.

A systems-oriented perspective is particularly useful in the context of agricultural innovations and their governance systems, where technological change is influenced by an array of social, cultural, political, environmental, and economic factors. Agricultural innovations are inherently normative and embedded within existing value systems and cultural norms (Klerkx et al., 2012). Sustainable governance of these novel technologies requires an understanding of how these various factors interact (de Boon et al., 2022). Similarly, governance systems are not value-free. Policy and regulatory decisions are shaped by implicit worldviews, cultural norms, and institutional priorities (Pascual et al., 2023). For example, in the environmental assessment of GE crops, the selection of parameters and risk criteria are shaped by broader societal influences and assumptions (Kuzma et al., 2023).

Systems-thinking is also a major component of several established upstream frameworks that are discussed in the governance of biotechnologies: Responsible Innovation (RI), Safe-by-Design (SbD), and the Safe(r) and Sustainable Innovation Approach (SSIA). These frameworks collectively shift responsibility from reactive regulation to the early design and development phases. RI, as defined by Stilgoe et al. (2013), emphasizes four dimensions, anticipation, reflexivity, inclusion, and responsiveness, for a system level analysis of an emerging technology. Similarly, SbD integrates broader safety considerations into the earliest stages of innovation (van de Poel and Robaey, 2017). Finally, the OECD’s SSIA framework bridges these concepts by combining SbD with “regulatory preparedness” to align innovation with safety and sustainability requirements from the outset. With these frameworks pointing to the emergence of systems thinking’s influence on the regulatory landscape, it is critical to assess the degree to which systems thinking influences the actual practice of regulatory science.

Focusing on the U.S. context, this study investigates how different stakeholder groups (e.g., academia, industry, government, NGOs) perceive the regulatory and oversight landscape for GE crops and emerging biotechnologies. It further examines how these perspectives inform interpretations of existing oversight processes and influence views on assessments of both existing technologies like traditional genetic engineering (e.g., transgenesis) and emerging innovations such as (cisgenic) gene editing. Participants were also invited to share their visions and recommendations for the future of environmental oversight in this domain. By analyzing diverse viewpoints, this study aims to illuminate how perceptions among these differing stakeholder groups shape the environmental oversight of biotechnology in agriculture. Accordingly, the systems-oriented grounding informed how interview questions were developed as well as how interconnections, boundaries, and cross-level dynamics were interpreted during analysis.

### 2.2 Participant recruitment

A total of 100 potential participants were initially identified across stakeholder groups including academia, the biotechnology industry, government (including recently retired or transitioning professionals), and NGOs based in the United States (U.S.). This was done through a systematic search of peer-reviewed publications, reports, databases, and conferences, as well as through professional contacts and networks. This purposive sampling strategy (Campbell et al., 2020) targeted experts in GE crop oversight, environmental risk assessment, regulatory policy, and biotechnology governance. The initial recruitment email also included a request for recommended additional contacts, yielding 20 additional potential participants.

Of the 120 individuals contacted, 16 agreed to participate. Participants represented the four stakeholder groups (academia: *n* = 4; industry: *n* = 5; government: *n* = 4; NGO: *n* = 3) and held roles ranging from researchers and risk assessors to domestic and global policy leads. While these roles were not analyzed separately, they helped verify that all participants possessed relevant expertise. Once the 16 interviews were completed, sufficient thematic saturation (in both depth and diversity; Yang et al., 2022) was achieved and thus no further participant recruitment occurred.

### 2.3 Data collection and interview process

Semi-structured interviews were conducted, where the interview protocol was developed *a priori* influenced by the aforementioned systems thinking approach (Adams, 2015; Galletta, 2013). The interview protocol was submitted to the North Carolina State University Institutional Review Board (IRB) and was deemed exempt from further review (Protocol 28215). Participants received the interview questions in advance, along with informed consent materials. They were also provided with definitions of genetic engineering and gene editing to ensure a shared understanding of terms, as multiple definitions are used across stakeholder communities (Grieger et al., 2024a; NIST, 2025; Nordberg et al., 2018). The definitions provided to study participants included:

- *Genetic engineering*: refers to a set of techniques designed to change or alter the genetic makeup of an organism by introducing, removing, or modifying specific genes.
- *Gene editing*: refers to a relatively new genetic engineering method that can edit an organism’s genetic material and does not necessarily involve using another organism’s DNA.

Interviews were conducted *via* Zoom between July and September 2025 and lasted an average of 45–60 min. The interview protocol consisted of nine total questions: one introductory question to establish the participant’s background and eight questions aligned with the study’s three research questions:

1. What are stakeholders’ perceptions and views of the environmental assessment and oversight processes for genetically engineered agrifoods?
2. In what ways do differing perceptions influence the interpretation and regulation related to environmental oversight as it relates to emerging biotechnologies, such as gene editing, and how are these technologies understood in relation to existing GE oversight frameworks?
3. What visions or recommendations do stakeholders offer for improving or reimagining the environmental oversight process for GE and gene-edited crops?

Table 1 presents the structure of the interview guide, the purpose of each question set, and their alignment with systems thinking and the research questions. Some questions also included sub-questions to explore specific concepts in greater depth. Interviews were audio- and video-recorded and transcribed through Zoom’s integrated transcription tool; transcripts were subsequently reviewed and corrected by the primary researcher to ensure accuracy.

**TABLE 1:** Interview structure and alignment with systems thinking. illustrates the semi-structured interview guide. It summarizes how the interview questions were organized and how they align with the research questions and systems thinking.

Section	Purpose	Systems thinking alignment	Research question	Interview questions
Background	Establish role, orientation	Stakeholder position in the system	RQ1	1. Could you tell me a little bit about your background?
Assessment process	Understand technical and experiential views	Identifies strengths, weaknesses, and boundaries	RQ1, RQ2, RQ3	2. How would you describe your understanding of the current environmental oversight process for GE crops in the United States? 3. In your view, are there specific environmental or ecological data gaps that hinder comprehensive environmental assessments, if any?
Emerging tech	Examine continuity/disruption in oversight	Adapts to evolving complexity	RQ1, RQ2	4. As you may know, there are newer techniques available to develop GE crops, such as gene editing techniques like CRISPR. To what extent is the existing environmental oversight framework sufficient for evaluating crops produced with these novel technologies? 5. What are your thoughts on current regulatory approaches to gene-edited crops that are exempt from certain oversight requirements? 6. From your perspective, what are the broader social, environmental, or economic stakes involved in the effective regulation of GE crops?
Recommendations	Elicit future visions and change levers	Points to system-level interventions	RQ3	7. How can regulatory agencies address emerging genetic engineering techniques and novel GE crops effectively? In an ideal world, what would the environmental oversight regulatory process of GE crops look like?
Final thoughts	Surface unanticipated insights	General systems awareness and anticipating future issues	RQ1, RQ3	8. Are there any other issues or concerns regarding the environmental assessment of GE crops that we have not covered but that you think are important?

### 2.4 Analysis

All 16 transcripts were de-identified and analyzed qualitatively using the software program Dedoose. The analysis began with open coding in the exploratory phase. This included an inductive coding process to identify initial meaning in the data through developing and applying descriptive codes (Bingham and Witkowsky, 2021). This first step generated 164 initial codes. The array of codes were organized into a select number of groups based on emerging, researcher-identified patterns that would later be characterized as the parent codes in the analytic process. A codebook was created to document each parent code, its associated child codes, and illustrative excerpts from across the transcripts.

Following this organization, codes were iteratively refined into broader themes and subthemes. Throughout this process, attention was given to the relationships between codes, system boundaries as defined by participants, and multi-level influences described by participants, consistent with the systems thinking framework guiding the study. To strengthen credibility, several peer-debriefing sessions were conducted with experienced qualitative researchers throughout both the research design and coding phases. Peer debriefing, an established qualitative rigor strategy, helped identify potential researcher bias, validate coding decisions, and ensure analytic rigor (Lincoln and Guba, 1985; McMahon and Winch, 2018).

### 2.5 Reflexivity and positionality statement

The research team for this study encompasses a wide interdisciplinary background in risk governance and systems-oriented perspectives. This study is heavily informed by the findings of prior research that advocated for stronger environmental oversight of biotechnology (Loschin et al., 2025). Acknowledging these preexisting views, the research team continuously engaged in reflexivity, questioning the assumptions and potential biases throughout the research to try and best understand how they inevitably shaped the study design, interview process, and data interpretation.

In general, participants expressed confidence that existing environmental oversight for GE crops is effective, with a strong consensus that direct ecological risks are well-managed under current regulations. This emergent consensus, shared across diverse sectors, at times stood in contrast to the primary researcher’s initial critical view that the governance system might be insufficient. Recognizing this divergence, the primary researcher consciously examined how [their] own critical lens could affect data interpretation. During analysis [they] took care to ensure that thematic coding and conclusions were grounded in participants’ actual statements and viewpoints, rather than being steered by the researcher’s prior expectations.

As this research was part of the primary author’s dissertation research, [they] engaged the other authors in peer debriefing to ensure interpretive rigor and to decrease potential bias in the interpretation of results. However, in qualitative research, it is nearly impossible to bracket all bias; thus, we provide an overview of the authors’ disciplinary and experiential perspectives that influenced subsequent analysis and interpretation. The primary researcher ([NL]) was a doctoral candidate at the time of the research, and is a interdisciplinary scholar focused on the environmental governance of biotechnologies [They] also worked as research fellow in the Office of Research and Development at the EPA, which likely influenced much of the aforementioned bias in the research. Additionally, the secondary author ([KS]), is an Assistant Professor and Extension Specialist whose work focuses on enhancing democratic dialogue and stakeholder engagement in the development of new and novel agri-food biotechnologies. Finally, the last author ([KG]) is the Director of the Genetic Engineering and Society Center (GES) at NC State University, among other leadership roles, who has specialized in risk assessment and risk governance of emerging technologies throughout [their] career. While qualitative research, as the current authors have operationalized it, does not seek generalizability but rather thick, rich description of participant perspectives (Lincoln and Guba, 1985; Yang et al., 2022), we offer the above subjectivity statement to invite readers more closely to the analytic and interpretive perspectives that influenced this study.

## 3 Results

Results are organized by the three major themes identified from the stakeholder interviews: (1) *the role of the environment*, (2) *the regulatory system works–mostly*, and (3) *new and emerging technologies in the current system*. Each theme also has additional subthemes as well as recommendations expressed by participants related to the respective theme. All participants were assigned an identifier using the following convention: “P1, Sector,” in which “P” stands for participant, “1” refers to the order in which participants were interviewed (1–16), and “Sector” refers to the sector in which they worked or previously worked (industry, government, academia, and non-governmental organization [NGO]).

### 3.1 The role of the environment

As this study focused on stakeholder perceptions of environmental oversight in particular, *the role of the environment* served as a central component of the research design in order to narrow the scope of the research questions. Because of this, environmental considerations were intentionally embedded throughout the interview guide to explore participants’ perceptions of the adequacy of environmental assessment for GE crops and to capture diverse viewpoints on potential ecological impacts. As one participant summarized:

I’m always one to say, you know, nothing is going to survive in agriculture, if it is in the end going to harm the environment, like there’s always going to be a ramification back to agriculture. Whether it is about a water quality problem or whether it is about the functioning of the farm, or whether it is about superweeds, or whether it is about public resistance because of overly non-precautionary approaches, we give the environment short shrift. But it is in fact what sustains us all, and we ought to be paying attention to that also. So it is not just what happens on the field. It is also what could happen off the field. (P2, NGO)

Participants described the complexities of environmental concerns for GE crops, noting the broadness of the term “environment” and how it may confer different meanings to different individuals as well as the uncertainty and longitudinal data needed to evaluate impacts. Notably, discussions frequently expanded beyond direct environmental effects of GE crops; in other words participants often discussed more of the broader evaluations of the overall regulatory system or toward environmental implications of other emerging agricultural biotechnologies rather than detailed responses to environmental assessments. For example, one participant stated:

When we think about biotechnologies in the narrow mindset of engineered crops or other kinds of organisms, we also forget that biotechnology is literally any life form that we put to use for human practices. It is made up of the stuff and the similar kinds of will to live as us. So that’s why, like socioculturally, it is really complicated, the connection between regulation and that stuff. When we think about doing harm, we have very particular ways of thinking about that from a regulatory standpoint. The risk to usually just the physical wellbeing of human beings. Right? That’s how we think of harm and hazard. But if we expand that, maybe more to like socioeconomic damages and take that seriously, it’d be interesting to see what governance looked like. (P5, Academia)

With these insights, two subthemes emerged: (1) reducing the emphasis on the environment, and (2) direct and indirect environmental concerns related to agricultural biotechnologies.

#### 3.1.1 Reduced emphasis on the environment

Despite the study’s explicit focus on environmental issues, most participants did not anchor their responses in environmental impacts, as briefly mentioned above. Instead, they gravitated toward broader concerns about the regulatory system’s structure and functioning (see Section 3.2). Many participants expressed confidence in the environmental safety of GE crops in controlled agricultural systems and did not view environmental impacts as a primary concern. For example, P15 (Government) explained, “I do not think environmentally there are big issues. It is farming,” which in this context alluded to the idea of farming being a controlled, confined process. Additionally, P5 (Academia) stated, “I think, in the crop space [environmental oversight] might be okay. If the crops are not able to propagate and colonize other spaces.” Another participant noted:

Anything that could have a potential to harm the environment is eliminated early from the pipeline and is not actually advanced forward, because we have criteria, not only technical criteria and efficacy criteria, but also regulatory criteria that eliminates what I would call the traits that could present an unacceptable risk. (P6, Industry)

Because GE crops are typically deployed in managed agricultural settings, many participants perceived environmental risks to be well-characterized and adequately mitigated under the existing assessment processes. Instead, participants tended to focus on regulatory burden, broader ecological interactions, or issues arising from other agricultural biotechnologies.

#### 3.1.2 Direct and indirect environmental concerns

Among the stakeholders who did discuss environmental considerations, their concerns centered less on direct impacts that are typically evaluated by the EPA and USDA (e.g., gene flow, weediness, impact on non-target organisms) and more on the indirect environmental impacts. These included concerns about environmental persistence and ecological interactions as one participant stated: “This issue of persistence is something that is more novel as we are looking at putting things in the field, in the forest, in water systems, and soil systems that are designed to persist.” (P5, Academia, emphasis added), such as GE microbes. This sentiment was further expressed by another participant when thinking about emerging biotechnologies: “We are releasing organisms into the environment for the stated purpose of having them disseminate into the environment rather than keeping them contained in an agricultural field or in the laboratory, or somewhere else.” (P2, NGO, emphasis added). Here, indirect impacts refers to effects that arise not from the GE crop itself but from how a trait interacts with the environment and biological systems. For example, one participant questioned the inconsistency between regulating plant-incorporated pesticidal traits in GE plants and the use of the same traits in microbial biopesticides:

We already have these bacterial strains that are sprayed on crops. So the same traits that are in the bacteria are sprayed on plants. We only put one or two or three genes from these bacteria in plants, so you already have this technology out there somehow in the environment, and one is exempt from regulation. The other one is regulated every single time. When you talk about the environment, **why are you not worried about spraying, which actually spreads it in the environment** (P6, Industry, emphasis added)

While this perspective reflects stakeholder concerns about comparative environmental exposure, it is important to note that microbial biopesticides are not unregulated; rather, they are evaluated under differing EPA frameworks compared to plant-incorporated protectants, illustrating the potential inconsistency in assessment for the same trait.

At the same time, the conversations revealed an emerging need for broader, systems-oriented approaches that can account for indirect impacts and the expanding landscape of agricultural biotechnologies. One participant expressed the lack of environmental assessment when thinking about the impact on natural systems:

Nobody has been on point to really think about **impacts in natural systems**, of escapes or of gene flow that was not intended and so that has always been a big hole in this system, and one that just sort of confounds me. I think that the system itself is sort of flawed because it is process-based rather than product-based. I understand process-based as a first cut because you have to organize in some way, but really we should not be thinking GMO, or not GMO, we should be thinking that this GMO, or this gene-edited, or this bio product, what are its potential benefits, what are its potential risks (P2, NGO, emphasis added)

While gene flow is considered on a case-by-case basis by the EPA and USDA for crops they have jurisdiction over, such examples illustrate how participants broadened the discussion beyond GE crops to question the oversight of agricultural biotechnology more generally. Overall, stakeholders expressed strong consensus that direct environmental impacts of GE crops are well-managed, while indirect impacts and emerging technologies remain more difficult to assess within the existing framework.

#### 3.1.3 Recommendations

Participating stakeholders who expanded their environmental considerations beyond direct impacts offered recommendations that emphasized holistic, systems-level evaluations. A government participant described how current assessments remain segmented across agencies and fail to capture interactions between traits, crops, and agricultural practices:

The idea is that we just approve the crop and say, is it a noxious weed or a plant pest? They do not look at, okay, that’s going to increase herbicide use. I mean, again, APHIS looks at it in its Environmental Impact Statement, but they do not do anything about it. I mean, they give some analysis. This will increase glyphosate use or dicamba use, or something like that. EPA looks at glyphosate and says, well, glyphosate is going to be used on these different crops. Are there risks associated with that? But we do not put those together and **take a holistic look at that crop and that trait, and the agricultural system** that is going to be introduced into. (P3, Government, emphasis added)

Another participant advocated for considering ecological community dynamics, especially where biotechnologies are intended to modify environmental conditions:

Meaning, **how does this organism, once it is inserted into a particular environment, change that environment?** Because in the agricultural setting, in the environmental remediation setting, you would want to have an organism like a bacterium that’s maybe cleaning up a chemical spill or doing some sort of nitrification or phosphorylation work in an agricultural field to restore soil fertility. You’d want that organism to exist for a while to do the work that it needs to do and you want it to change the dynamics of that soil. You want to detoxify, you want to insert more nitrogen soil. That’s kind of a different paradigm of thinking than you know, insert a crop into a field, it does work, and then it is gone, and you know the field’s the same as it was. **We’re now using crops and microbes to change environments.** So that’s definitely new. I think that’s a paradigm shift. (P5, Academia, emphasis added)

Both of these recommendations point toward a more holistic environmental assessment approach to fully comprehend the ramifications of existing and novel biotechnologies in agriculture. However, participants also acknowledged that implementing such systems-level assessments would be challenging within existing statutory boundaries.

### 3.2 The regulatory system works–mostly

The second major theme from the interviews was that the *regulatory system works–mostly*. This theme encompasses participating stakeholder perceptions of the structures and processes of the U.S. system, as well as the scientific and risk assessment approach for governing GE crops. The consensus among the interviewed stakeholders (e.g., government, academia, industry, NGOs) is that when looking at environmental safety, the current system is generally effective. Participants across stakeholder groups consistently described the U.S. system as robust enough to prevent unsafe GE crops from entering the market:

I would say, generally, if I’m talking about purely environmental assessment, I think it is pretty good. Because, again, basically, what they did is, they took the chemical regulations, and they adapted them to [GE crops. I would say, for the first event, it is very good, meaning that it will prevent any rogue companies from putting something on a market that they should not put on a market, and that’s why the safety profile for biotech is excellent.” (P9, Industry)

This sentiment was also shared by a government participant: “I think it is pretty robust in that nothing has really broken the system yet. No GE crop has broken the system yet.” (P13, Government). As well as a participant from an NGO: “Yeah, I do think there are not very many examples yet of where there have been inadequate risk evaluations before permission to release. (P2, NGO) and a participant from academia:

I do feel that the existing framework is reasonable for the hazards that seem to be of concern. Human health effects, biological invasions, I do not know what else, really. I do not think those are huge risks. I think that the screening that we have is reasonable for those. (P16, Academia)

Despite this shared confidence in environmental safety, participants expressed more concern about regulatory burden, statutory limitations, and the challenges of fitting new technologies into outdated frameworks.

#### 3.2.1 History of safe use

The most frequently referenced justification for confidence in the system was the *history of safe use*. Participants emphasized that over 30 years of cultivation for GE crops in the U.S., there has not yet been direct evidence of direct environmental risks. Industry stakeholders in particular described how decades of field data have helped refine regulatory expectations:

Very early on, when we were doing everything from the lab in the field. **The field work was a nice validation of what we found in the lab**, so it provided nice confirmatory information. But as time has gone on, we’ve demonstrated a history of safe use with genetically modified crops. The regulations have evolved and become more streamlined in some areas of the world. Not all world areas. But certainly it becomes streamlined again. Learning as we’re going, based on experience, you’d expect a natural evolution in that area. We’ve got more information, more studies. You can update your regulations. (P1, Industry, emphasis added)

This history of safe use is also central to evaluative practices, where regulators compare GE crops to non-GE counterparts. Several industry participants advocated for stronger integration of this principle, arguing that oversight should be proportional to known risks and exposure pathways:

I’m not saying that we should be reckless. I think that there should be some oversight, but that oversight needs to be **based on proportionally the amount of risk and exposure, and science and the history of safe use** that we definitely established by now, if not for all of the traits, but at least for some of the traits. It works the same, and we have not seen any surprises that way, so we should take some steps to make it proportional to the risk exposure. (P7, Industry, emphasis added)

However, not all participants viewed reliance on historical experience as sufficient. One academic stakeholder cautioned that familiarity can lead to complacency:

I do recognize that there are always going to be unintended risks, for just about anything, and we should be thoughtful about it, but I think we’re being a little bit cavalier about some of these older technologies, just because they’ve been around for a while, and we do not think about how they can present risks, too. (P11, Academia)

Questions also arose regarding how regulators use history of safe use in decision-making. One participant expressed frustration with the transparency and rigor of the USDA’s Sustainable, Ecological, Consistent, Uniform, Responsible, Efficient (SECURE) Rule.

I’ll be honest, from what I’ve learned about the USDA SECURE process. I was not super impressed to be real with you. The process itself just did not seem overly rigorous. Kind of like, please give me some feedback on this, on this perspective. But it seemed like, oh, it is been done before. Great! Oh, we have like a couple of studies. And you know that there’s a problem with that RSR process, the lack of transparency, but not just the transparency of what’s gone through, but how the heck are they actually assessing the literature that exists around certain organisms and deciding on no plant pest risk (P5, Academia)

Although USDA has since returned to the *Am I Regulated* process, where developers may submit an inquiry on if their modified organism is regulated, similar concerns remain. Overall, while the history of safe use is a widely accepted strength of the system, it also raises questions about transparency, rigor, and potential over-reliance on familiarity in the regulatory process for agricultural biotechnologies. Participants’ critiques of the regulatory system were focused on statutory limitations and regulatory burden, which shape what agencies can evaluate and how companies navigate the system.

#### 3.2.2 Statutory limitations

When participants discussed the strength of the system, it was often caveated with the idea that the system has been successful within the boundaries set by the existing statutes that the USDA and EPA use to justify their jurisdiction over a GE crop. For example, EPA’s authority over Plant-Incorporated Protectants (PIPs) derives from Federal Insecticide, Fungicide, and Rodenticide Act (FIFRA) and applies only to pesticidal traits, not whole-plant interactions. Similarly, USDA’s authority under the Plant Protection Act is limited to plant pest harms. One former regulator summarized the challenge:

I think they’re fundamentally missing the issue. It is not an issue of coordination. **It is an issue of legal authority**, I mean, you can coordinate as much as you want, but as long as the lawyers say, the statute only allows us to do this, or we have to do this. You’re still gonna have overlaps and authority and duplications of work and missing work and things like that. It is the natural evolution of fitting square pegs into round holes, putting new technologies into old statutes that do not fit really well. And so you’re always going to have gaps and ambiguities and overlaps, and you can coordinate the hell out of that, as all you want, but until there are changes in the mandates, it is going to get thrown against sort of these brick walls. (P3, Government, emphasis added)

Participants noted that emerging biotechnologies challenge the existing CFRB often blurring the lines of jurisdiction:

The one thing that could be improved upon is the coordinated framework [CFRB]. I think the more science advances, and the more novel products that we see, the more questions come up about regulatory jurisdictions, and is this a plant pest, or does this matter (P13, Government)

#### 3.2.3 Regulatory burden

The second major critique among participating stakeholders concerned the regulatory burden. Issues were raised on the cost, complexity, and length of approvals when navigating the approval process. Participants described the burden as especially difficult for small developers:

Regulatory costs for developers of genetically engineered crops had been quite steep. And while big companies can easily absorb those costs, smaller companies, startups, have a much more difficult time absorbing those costs, because they end up being a much larger percentage of a small startup’s company’s budget than it does for a large company. So, it can be really problematic for a small startup when all the technologies are tied up in patents. (P15, Government)

One academic who has worked with various companies shared the importance of understanding the regulatory process:

What we’re finding in our work is that the difference between successful companies and unsuccessful companies is whether or not they have a big difference. Besides, like, you know, being able to create a technology that works is, **do you have someone on staff who’s an effective regulatory affairs person who knows how to navigate those complexities and regulations**? And more than that, more than just the knowledge aspect. Do you bring them into strategic thinking early on in your business, and you’re informing your business plan and developing and designing your technology (P5, Academia, emphasis added)

Even large, well-established companies described extensive timelines and escalating requirements. A former regulator discussed the phenomenon as oversight creep:

I think, in general, the oversight has, I think, there’s been oversight creep, since regulations were first put in place, or since agencies started regulating genetically engineered crops. I think there was some thought, looking back historically, and having some discussions with people at APHIS, particularly. There was some thought that they’d regulate things for a while, 5 years, 10 years, 15 years, something like that. And then, as people became comfortable with the technology and such, the regulations would sunset. And go away, but that never happened. **As with many regulations in the U.S. government, there tends to be mission creep, there tends to be regulatory creep,** and at some point, everybody feels they need to have a say in things. (P15, Government)

Participants across sectors raised concerns around the convoluted nature of the regulatory process and how it has led to disproportionately extensive data and testing. Questions were raised on the need of these long-standing data requirements given the history of safe use for GE crop safety:

I think, in terms of where it could need improvement, it is largely that a lot of our processes were sort of developed 30 years ago, when these GE crops were brand new, novel products, and people were squeamish or nervous about it. I think now it is sort of time for us to take a look at the processes that we’ve had. **What are the data that have been required? Do we really need those as empirical studies?** Or can we start to rely more on rationale, and in terms of the work at our agency, also? What are some of the restrictions that are in place, or additional regulatory burdens that might be able to be eased? Now that we have, you know, 30 years of experience working on these types of crops. (P8, Government, emphasis added)

P6 furthered this sentiment stating:

Maybe now it is time to start looking at **are some data points really needed**? If the traits are still the same, just a different iteration of an insect control, or in a different iteration of herbicide tolerant, or a different iteration of another of these traits, do they really actually affect significantly the risk of going down in the environment? And the answer is typically no. (P6, Industry, emphasis added)

These comments highlight a shared perception of a system that is unnecessarily burdensome and may require a reframing of the assessment approach to better streamline approvals based upon decades of experience and data collected on GE crops.

#### 3.2.4 Recommendations

Participating stakeholders proposed two areas of improvement for the regulatory process: (1) centralized and coordinated oversight, and (2) risk-proportionate assessment frameworks.

In terms of having a more centralized and coordinated oversight process, participants frequently described fragmented agency responsibilities as a barrier to efficiency and consistency. Concerns were raised around the lack of coordination and repetitiveness among the three agencies for biotechnology regulation (USDA, EPA, FDA). Several stakeholders suggested a centralized coordinating office, not necessarily a full agency, to streamline the assessment and approval process:

I’m just thinking that there needs to be a coordinating office. I think that the **coordinating office could help with addressing overlaps between different agencies**, regulatory authorities, and how they deal with those products that come to multiple agencies. Such as making sure everyone uses the same definitions. That seems basic, but it is not. Yeah, I do not know, those are some things in terms of coordination. (P12, NGO, emphasis added)

P13 suggested something similar:

Instead of parsing things out into these three agencies, maybe it would be more effective and efficient if you could just submit something to one agent, one group, and figure it out that way. Even if you had one group where developers could go and get some immediate feedback, they could say, well, you only need to submit this data, that thing. It would be very obvious that would be the need, but right now, developers have to do these things on their own, figure these things out themselves. (P15, Government)

A centralized office was also seen as a way to enable more proactive governance for the influx of emerging biotechnologies, compared to the current systems in place at specific agencies:

This office is the proactive thinking body that then informs EPA work. And you see that as more efficient than using, I mean, what the EPA is probably utilizing right now, and they’ll do it in their justifications for their reviews, is, you know, sciences, boards, advisory panels, and science advisory boards where they’re putting together the academics. It is not something that happens often. So it is like, I guess it is not proactive. Because, yeah, it is happening. I do not think scientific advisory panels are the move to engineer comprehensive governance of emerging biotechnologies. (P5, Academia)

In terms of having more risk-proportionate assessments, participants also emphasized a risk-proportionate regulation system that balances oversight rather than uniformly applying legacy requirements:

My big takeaway to you for all your questions is, we should have **oversight that matches** the **potential risks** of the technology. What are the potential risks of any products of a particular technology? And then what can we do to assess and manage those risks and link the exposure pathways and the potential risks to the assessment that we do, as opposed to assessing things that may or may not ever be a real risk for a particular technology (P3, Government, emphasis added)

In this risk-proportionate framework, several stakeholders also encouraged incorporating benefits into evaluations, which is something only the EPA is mandated to do under FIFRA and does not apply to USDA decisions for GE crops. Many participants discussed the importance of weighing the benefits such as:

Yes, there may be some risks, but do the benefits outweigh it? If this product cannot be used, it is going to affect, say, wheat yield and production in Montana by 30%. And this is the only herbicide that can control that specific weed in the crop and without it there’s going to be huge economic implications. Then you gotta play that game of **balancing the risk and the benefit**, and again finding risk, mitigation or management solutions, if possible. (P1, Industry, emphasis added)

Together, these recommendations reflect a desire for a more comprehensive regulatory approach that balances the potential risk and benefits of a product while reducing unnecessary burdens and streamlining the approval process.

#### 3.2.5 Systems orientation - informing critiques

Collectively, participants’ reflections on statutory limitations, regulatory burden, and fragmentation reveal a broader systems orientation in how they view regulatory challenges. While participants generally expressed confidence in the environmental safety evaluations conducted by USDA and EPA, they also highlighted that the system is increasingly strained by novel biotechnologies, overlapping jurisdictions, and outdated statutory foundations. This systems-level critique does not undermine participating stakeholders’ confidence in existing assessments for GE crops. Instead, it shifts the perspective from solely on the product to a holistic view of the agricultural system, offering a reflection on some of the historical and current gaps and how these novel applications for biotechnology will enter these already strained spaces.

### 3.3 New and emerging technologies in the current system

The final major theme gleaned from the study was around how *new and emerging agricultural biotechnologies fit within the current U.S. regulatory system*. While participants expressed general confidence in environmental oversight for GE crops, they raised concerns about the system’s capacity to evaluate technologies that depart from traditional plant breeding and genetic engineering paradigms. Much of the perceived regulatory burden was associated with the pace of novel innovations and the outdated regulations that are struggling to keep up with these changes.

The interview guide included questions about the oversight of novel products, with the focus on some of the NGTs, such as gene-editing in crops. However, participants overwhelmingly did not express concern for any unique risks to these gene-edited crops and deemed them similar to conventional breeding. One industry participant summarized this view:

Our long-term goal is to get our agencies comfortable enough to the point to say, look, these are no different from conventionally bred crops. The risk is the same as a conventionally bred crop. You have laws on the books that allow you to regulate them in or capture them, somehow, if they do end up causing a problem. And if products are kind of equal, then they should be treated the same under the law. (P14, Industry)

Several participants emphasized that gene editing (particularly cisgenic applications) offers a more targeted approach and fewer unintended changes compared to first generation transgenics:

The issue here is that we have fewer off-targets and a better sense of what we’re doing. So I think there’ll be fewer surprises. I am less concerned in general about genome-edited crops than about the early transgenics. We have better control over where things get into the genome, and a better sense of the copy numbers that go into a site. (P4, Academia)

In contrast, participants expressed significantly more concern about emerging biotechnologies that fall outside the conventional GE crop paradigm, including gene drives, engineered microbes, and applications in synthetic biology. These technologies were viewed as especially challenging to regulate under the current system:

My bigger worry is that the process would be rehashed and reformulated to work, let's say, for genetically engineered microorganisms, which would be a terrible system. Absolutely the wrong move, and in an instance where there is not a lot of resources or time on the hands of EPA professionals, and if they're the ones that are playing a big role in regulating … environmentally engineered microbes for environmental release, then they're like, Oh, we need to come up with a system. Let's see what else has been done. And I worry that basically a copy-paste of a system like that would come out for genetically engineered microbes. (P5, Academia)

These concerns highlight a finding from the study that these emerging biotechnologies beyond GE crops raise questions about the plausibility and nature of potential environmental harms, which should be assessed before determining the appropriate oversight and assessment approach going forward.

#### 3.3.1 Novelty considerations based on trait and impacts

When discussing emerging technologies, participants constantly reiterated the history of safe use for GE crops, but the importance of case-by-case evaluations based on novelty of the introduced trait and the potential environmental impacts. As one participant explained:

That’s why, the first of a kind always gets more oversight, because they’re new and different and novel, as you get to know things more, much more comfortable with them. They get fewer levels of oversight. And so the level of comfort we have with one, the level of familiarity you have with something, is always going to balance into how much oversight that should go to. The more novel what you’re doing is, and the kind of organism you’re using, and so forth, and so on, should go into factoring whether it gets a cursor, review or no review. (P3, Government)

Participants discussed the need for trait-based oversight, as is prevalent in the product *versus* process discussion, noting the challenges of implementing it into the current system:

In an ideal world, we would be doing this based on the traits that crops have. It is not simple to create a system that way. It is a lot easier to be like this type of genetic change, or not, like, we sequenced it, we can look at it. It is a little blurrier to use traits, but I think it would be a lot more effective at matching pre-market reviews with proper resources with the riskiest products. (P12, NGO)

Some participants expressed going beyond novelty considerations to also understanding how a trait interacts within agricultural systems. One government participant illustrated this point by highlighting the interdependence of crops, traits, and management practices:

Well, I think, for example, **we cannot think of a trait without the way that’s utilized within the agricultural system**. So the idea that our oversight of genetically engineered herbicide-tolerant crops just looks at the crop, but does not look at the crop trait, the crop herbicide combination, seems to me unusual that you, you know, we should not just look at. (P3, Government, emphasis added)

This holistic perspective allows the conversation to go beyond the product *versus* process debate to broader systems-level view to further understand the ecological, agronomic, and socio-environmental context of emerging biotechnologies.

#### 3.3.2 Recommendations

In order for the regulatory process and framework to keep pace with these novel innovations, participants proposed two key recommendations: *proactive regulation* and *adaptive management*. First, participating stakeholders expressed concern that the regulatory bodies tend to act retrospectively and after a novel product is developed and ready for market approval. Several participants advocated for anticipatory governance practices, especially for novel emerging technologies beyond traditional GE crops:

So, building new marketplaces for emerging biotechnologies will inevitably require the involvement of regulators and how regulations are constructed … this kind of dictates which pathways are feasible for companies, in certain ways, and I think that as proactive as we can be the better. Which means regulatory agencies like the EPA, have to start thinking about genetically engineered algae for carbon sequestration right now. Which is weird because it's basically saying, we want your office to do some work on this 20 years, maybe before it might actually enter into the field, and everything I've come to know about the EPA process is something might not be thought about until it's on the desk, and it's like we are a company who wants to deploy this as soon as possible, which I think is too late to think about good governance for emerging biotechnologies. (P5, Academia)

This forward-looking perspective aligns with larger critiques of existing statutes and was closely tied to recommendations in Section 3.2.3 for a centralized, biotechnology-focused coordinating body. Additionally, participants also emphasized the need for adaptive management as an iterative systems approach that updates oversight based on new data, monitoring outcomes, and emerging evidence. One academic participant defined it:

There should be meta-analyses done later to re-review this. Now, the way USDA does this, is they deregulate something and then it is done. Yeah, well, do we ever go back and see? Did we make the right decision? Are we practicing what we call adaptive management (P4, Academia)

Participants suggested that post-approval monitoring and periodic reassessment could help identify unexpected impacts and better align regulatory resources with emerging uncertainties. For example, improved monitoring for the GE crops currently in cultivation could be used to better understand the impact on the environment and inform future decision-making. As one participant said:

If [monitoring] is not happening anywhere, then you can get to a point where something’s being used on millions of acres across the country. And then, oh, crap. It is actually doing a thing we do not want to do, and nobody caught that. I think that’s a huge risk, especially now in this era of massive deregulation that we’re kind of entering into, or more fully like into now. If we’re going to do these kinds of interventions. The level of monitoring, like post-application monitoring, that would need to be done is not insignificant. Before you can know what you’re monitoring, you have to kind of understand that environment. (P5, Academia).

This evaluation of the system could be utilized to identify gaps in the process as well as identify opportunities to streamline the assessment approach. As more information and data becomes available, practicing a proactive and adaptive management system as part of the regulatory process can help focus resources and attention to more novel products and pacify the regulatory burdens that were expressed by participants.

## 4 Discussion

This study investigated U.S. expert stakeholder perspectives on the environmental oversight of GE crops and emerging biotechnologies through a systems-level lens. While there have been numerous studies published on the oversight and regulations of GE crops and agricultural biotechnologies (e.g., Buchman and Kovak, 2025; Loschin et al., 2025; Kuzma et al., 2023; Caradus, 2023b), comparatively little peer-reviewed research has systematically examined expert stakeholder perceptions of the oversight process itself (Kuzma et al., 2009). This study builds on the limited existing literature by focusing explicitly on environmental oversight processes, while also accounting for recent advancements in biotechnology, including gene editing and other novel applications. Moreover, this study aimed to explore participant perceptions regarding the current environmental oversight framework for existing and emerging biotechnologies as well as elicit any recommendations for improving the process. Based on stakeholder interviews, findings from this research gleaned three major takeaways related to: i) perspectives on the current oversight process, ii) considerations raised by emerging biotechnologies, and iii) recommendations for strengthening environmental governance of agricultural biotechnologies.

### 4.1 Processes for environmental assessment and oversight of GE crops are theoretically sound but narrow in scope

First, a central finding of this study is that expert stakeholders expressed strong confidence in the environmental safety of GE crops when placed in a controlled agricultural setting and grounded in more than 3 decades of cultivation without widely recognized direct ecological harm. This finding is supported by previous reports by NASEM (2016). Across the stakeholder groups interviewed, this rationale was rooted in the history of safe use and the perceived robustness of assessment for early transgenics and current GE crops. However, it also should be noted that the reliance on historical safety in closed systems could potentially narrow the scope of potential impacts investigated and when there may be a need for broader systems-based ecological considerations, an interpretation informed by the systems thinking framework guiding the research design. While there was an apparent reduced emphasis on the environment in many stakeholder responses, this did not reflect disinterest in environmental protection. Rather, the direct effects that are traditionally assessed by the USDA and EPA (e.g., gene flow, plant pest risk, impacts on non-target organisms) were widely viewed as well characterized and evaluated in current assessments of GE crops. In contrast, the participants highlighted the indirect effects of GE crops, such as persistence and impacts on biodiversity, as potential environmental concerns. Points raised by participants were focused on the introduced traits and use purpose of novel biotechnologies rather than differences in the genetic engineering techniques used, particularly when going beyond traditional GE crops and thinking about new applications such as GE microbes and gene drives that are designed for spread rather than containment.

Stakeholder participants also increasingly framed environmental risk as emerging from interactions among traits, crops, management practices, and broader socio-ecological systems. From a governance perspective, these interactions go beyond the product *versus* process conversations (Gould et al., 2022) and suggest the need for an environmental oversight paradigm that incorporates broader assessment endpoints beyond the evaluation of the product and the process in which it was created (Kuzma et al., 2023). A heavy reliance on historical safety may create blind spots when thinking about cumulative, landscape-scale, or long-term ecological effects of agricultural biotechnologies. For instance, a history of safe use does provide meaningful insight into the environmental safety of past, current, and future biotechnologies; however, it is limited in scope as emerging applications diversify beyond conventional cropping systems and compounded by a lack of retroactive assessment and adaptive management processes. While environmental risks are perceived as manageable in the current oversight process, they are often too narrow in scope when considering the whole system, a consideration informed by the study’s theoretical framework. A systems-level approach can broaden this scope, offering insight into the cumulative impacts that introduced biotechnologies may exert on natural systems.

### 4.2 Tensions between emerging biotechnologies and legacy regulatory frameworks

A second major finding of this study illustrates perspectives from stakeholders that discuss the scientific soundness of the current system while also recognizing the gaps and limitations in the governance process especially related to emerging biotechnologies. More specifically, participants across sectors consistently affirmed the technical robustness of environmental assessments carried out by the USDA and EPA within the bounds of their existing authorities. However, participating stakeholders emphasized that the current process was not designed for today’s biotechnology landscape and is increasingly strained by novel applications that blur jurisdictional boundaries. This observation is consistent with previous analyses noting that U.S. biotechnology oversight remains a patchwork of authorities dating back to the 1980s, which were never intended for some of the latest genetic engineering techniques (NSCEB, 2025).

Participants emphasized that coordination challenges for the environmental assessment of GE crops are less of a procedural failure and more related to trying to fit new technologies into old statutes. In particular, emerging agricultural biotechnologies enter a system with a large regulatory burden. Participants described long approval timelines, increasing data requirements, and oversight creep as major obstacles, especially for smaller developers. Interpreting these findings through a systems lens reveals that while participants focused on the immediate regulatory burdens hindering development, a systems perspective can help characterize these hurdles as an attempt to manage the complex trade-offs between rapid technological deployment and the prevention of long-term unanticipated environmental consequences. Finding a balance between enabling innovation and ensuring environmental safety is paramount for the future of emerging biotechnologies and addressing complex environmental issues.

### 4.3 Recommendations for strengthening environmental oversight systems for GE crops in the U.S.

The third key finding of this study is the set of recommendations proposed by participants. Despite being affiliated with different sectors (e.g., academia, government, industry), participants showed a strong convergence around a shared vision for the future of environmental oversight for GE crops in the U.S. The major governance needs proposed were creating (i) centralized and proactive oversight, (ii) risk-proportionate assessments, and (iii) adaptive management.

First, calls for a central entry point and increased coordination were centered around the need for a centralized body to streamline approvals and assessments as well as provide proactive guidance for emerging biotechnologies. This participant recommendation aligns with global practices for the governance of agricultural biotechnologies. For example, the European Food Safety Authority (EFSA) is a centralized coordinated body for the European Union that routinely publishes proactive horizon scanning reports for emerging biotechnologies and begins to think about how these novel applications will work in their existing systems (EFSA et al., 2025a; Ballester et al., 2023). Furthermore, the idea of proactive oversight and governance in a centralized ways is a common theme among upstream systems-oriented frameworks that can be applied to oversight settings, such as Responsible Innovation (RI) (Stilgoe et al., 2013), Safe(r) and Sustainable Innovation Approach (SSIA) (OECD, n.d.), and Safe-by-design (SbD) (van de Poel and Robaey, 2017) for emerging technologies. Establishing a more centralized and coordinated oversight body could allow for more proactive and forward-looking evaluations of emerging biotechnologies.

Further, risk-proportionate regulation was the most frequently expressed suggestion for improving the assessment process for GE crops and emerging biotechnologies among stakeholders. Participants argued that oversight should align with exposure pathways and hazard profiles rather than uniformly applying data requirements. They proposed that this approach should recognize the decades of safety data and streamline the oversight process. Some regulators such as the EPA implement this in their case-by-case approach where scientific consensus may be used instead of extensive laboratory testing (EPA, 2025). In turn, regulators then can focus their already limited resources on more novel applications and traits that are submitted for approval in the wake of NGTs and other emerging technologies. The recommendation for risk-proportionate regulations aligns with a systems approach by proposing oversight that should be tailored based on cumulative knowledge. Rather than treating every application as novel, a systems-oriented framework can help regulators recognize when a trait’s interaction with the environment is well-understood, allowing them to focus on uncertainties presented by truly novel biotechnologies. Risk-proportionate regulation integrates (and reflects from a participant perspective) a systems approach by reallocating regulatory resources from well-characterized cases toward emerging biotechnologies and applications where environmental interactions and uncertainties are greatest.

Finally, adaptive management emerged as a major recommendation for the governance of agricultural biotechnologies among stakeholders. Participants raised concerns over current regulatory decisions and the limited capacity for post-approval learning, reassessment, or iterative updates that may be needed after a product has been commercialized. This can be done with increased risk monitoring and management, especially if uncertainties arise with emerging biotechnologies, something that is done by EFSA with their annual Post-Market Environmental Monitoring (PMEM) reports and assessments for all GE crops (EFSA et al., 2025b). Given that existing regulatory structures are largely grounded in legacy statutes and can be slow to evolve in response to rapidly advancing technologies, an adaptive management approach offers a viable pathway for strengthening oversight of the next-generation of agricultural biotechnologies (Grieger et al., 2024b; Bennear and Wiener, 2019). From a systems-thinking perspective, adaptive management allows for a feedback loop to continuously strengthen environmental governance as applications expand into more dynamic and complex ecological contexts.

Together these recommendations illustrate a transition from the current static, front-loaded oversight process toward a more dynamic, and continuously adaptive regulatory system that evolves alongside emerging technologies (Ribeiro and Shapira, 2019).

### 4.4 Implications and future research toward a systems-level approach for environmental oversight

Strengthening environmental oversight of agricultural biotechnologies may depend less on refining the risk assessment process and more on reorienting governance toward a systems-level perspective, a view developed from the authors’ theoretical positionality as well as data gathered from participating stakeholders A systems-thinking approach can broaden the scope of oversight and consider how biotechnologies interact with ecological, agricultural, and institutional systems into which they are introduced. Furthermore, the focus shifts from how a product was made to how its proposed application interacts across ecological, agricultural, and institutional systems, balancing both risks and benefits across a variety of systems. This systems orientation provides a bridge between stakeholders’ confidence in current GE crop safety on the environment while offering the opportunity to reflect on the potential impacts that existing and novel applications of biotechnology have on a variety of ecosystems and natural systems. Participants’ recommendations for more centralized and proactive oversight, risk-proportionate assessments, and adaptive management reflect concrete points for integrating a systems-level approach. A centralized oversight body can function as a coordinating node across agencies, improving shared data infrastructure, horizon scanning for emerging technologies, and early identification of cumulative environmental effects across landscapes and regulatory jurisdictions. Integrating risk-proportionate and adaptive assessment frameworks would allow oversight to scale data requirements based on accumulated evidence, while using post-approval monitoring to create iterative learning mechanisms that refine future assessment thresholds and decision criteria. While certain responses from participants supported and reflected this systems thinking frame, gaps in their perceptions, particularly a tendency to view environmental safety primarily through the lens of individual product assessments rather than considering broader cumulative and cross-system impacts, indicate that more research is needed to fully explore and assess the potential for systems thinking’s role in the governance of agricultural biotechnologies.

Future research and additional stakeholder perspectives are needed to translate systems-thinking into actionable governance pathways. Comparative analyses of international oversight models may illuminate how other jurisdictions utilize anticipatory assessment, adaptive management, or centralized review mechanisms. Research examining how interagency coordination in the U.S. functions in practice, and where it breaks down, could further identify feasible points of institutional reform. Additionally, work is needed to develop systems-based assessment tools capable of integrating ecological interactions, cumulative impacts, and long-term environmental dynamics into regulatory decision-making. Environmental oversight of agricultural biotechnologies must evolve alongside scientific innovation and understanding, while being reflective of past experiences. The challenge remains in determining how such a holistic framework can be implemented in the U.S.

### 4.5 Study limitations

A few limitations exist in the current study and offer opportunities for future research. First, this study was limited to investigating stakeholder perceptions of oversight and assessment processes for GE crops within U.S. contexts. Therefore, findings generated are mostly applicable to the U.S. and may not be fully representative to oversight and assessment processes in other countries or regions. Additionally, due to the nature of qualitative research, the findings are not meant to be generalizable to all stakeholder perspectives; for this reason, interpretation should be bracketed to the study participants’ perspectives, despite having relevance and relation to broader stakeholder perspectives. Future studies could build on this work to focus on international contexts to further diversify stakeholder perspectives. Additionally, there were a total of 16 study participants, and while sufficient for this analysis due to thematic saturation, the study size could be expanded to include a greater number of study participants and from across different stakeholder groups - including those that hold different perspectives than the dominant viewpoints captured in this current work. In addition, this study was conducted in the middle of 2025, which was a time of significant change within U.S. federal agencies, which may have contributed to low participation of the 120 participants that were reached out to. Therefore, there were only two individuals who represented U.S. government agencies in this current work. Conducting a similar study in the future that included a greater number of participants from the government may offer new or different insights compared to what was captured in this work.

## 5 Conclusion

This study provides one of the few cross-sector qualitative examinations of expert stakeholder perspectives on the environmental oversight of GE crops and emerging agricultural biotechnologies in the U.S. Based on interviews with 16 stakeholders, findings reveal a regulatory system that is widely perceived as scientifically effective for traditional GE crops, yet increasingly strained by statutory limitations, regulatory burden, and the accelerating pace of emerging biotechnologies. This study advances systems-thinking approaches to biotechnology governance by demonstrating how expert stakeholders conceptualize environmental oversight as an interconnected system shaped by institutional boundaries, technological novelty, and socio-ecological factors, while also recognizing potential systems-level limitations presented by participant recommendations. Modernizing governance will require statutory innovations, systems integration across agencies, and new institutional capacities for anticipatory regulation.

## Data Availability

The raw data supporting the conclusions of this article will be made available by the authors, without undue reservation.
